# Retraction: JARID1B promotes metastasis and epithelial-mesenchymal transition via PTEN/AKT signaling in hepatocellular carcinoma cells

**DOI:** 10.18632/oncotarget.28847

**Published:** 2026-04-17

**Authors:** Bo Tang, Guangying Qi, Fang Tang, Shengguang Yuan, Zhenran Wang, Xingsi Liang, Bo Li, Shuiping Yu, Jie Liu, Qi Huang, Yangchao Wei, Run Zhai, Biao Lei, Hongping Yu, Xingyuan Jiao, Songqing He

**Affiliations:** ^1^Department of Hepatobiliary Surgery, Guilin Medical University, Affiliated Hospital, Guilin, Guangxi, People’s Republic of China; ^2^Laboratory of Liver Injury and Repair Molecular Medicine, Guilin Medical University, Guilin, Guangxi, People’s Republic of China; ^3^Department of Pathology and Physiopathology, Guilin Medical University, Guilin, Guangxi, People’s Republic of China; ^4^Department of Epidemiology and Statistics, School of Public Health, Guilin Medical College, Guilin, Guangxi, People’s Republic of China; ^5^Department of General Surgery, The First Affiliated Hospital, Sun Yat-Sen University, Guangzhou, People’s Republic of China

**This article has been retracted:** This decision follows an investigation by Oncotarget journal regarding the concerns raised by the readers on PubPeer. Our image forensics analysis revealed multiple issues of internal and external duplications and overlaps, and also reuse of the images of the same group of authors:

Internal duplications and overlaps:

- Figure 1B: b-actin western blot #1 was duplicated in blot #7.
- Figure 5C: Huh7-pBabe-JARID1B migration assay image overlaps with migration image siPTEN from different experiment in Figure 8H.
- Figure 5D: pBabe migration assay image overlaps with pBabe invasion image.
- Figure 5H: Migration assy image is partially duplicated in Figure 8H invasion assay of different experiment.
- Figure 8D: pcDNA-PTEN migration image overlaps with invasion image.

External duplications and overlaps:

- Figure1B: JARID1B western blot was also used as PTEN blot in Figure 9A of the simultaneously submitted article of the same group [1].
- Figure 3A: JARID1B western blot was found in Figure 5D as a-SMA from earlier paper [2] and as ZEB1 in [3].
- Figure 3G–3I: The colony formation assay images were found in Figure 5A from [4] and in [5], both published in 2014.
- Figure 3J: The colony formation assay images were found in Figure 2D of [1] and in [6].
- Figure 4A: The phase-contrast image of Huh-7 control cells was later reused by the same group in 2016 article [7].
- Figures 4A and 4D: The phase-contrast images were simultaneously published in Figure 2C and 2E of [8].
- Figure 4B and 4E: The images of immunofluorescent cells are duplicates of images in Figure 2C and 2E of [8].
- Figure 4C and 4F: The multiple western blots were duplicated in Figure 2D and 2F of [8].
- Figure 5B: The wound healing assay images are duplicates of images from reference [1, 3].
- Figures 5C, 5H and Figure 8D (corrected version): The transwell assay images were found in Figure 9C of [9], in Figure 5C of [10] and Figure 3H, 3I of [11].
- Figure 6G: The tumor images were earlier published in Figure 3A of [5], Figure 3A of [12] and Figure 2G of [13].
- Figure 7E: H3K4m western blot was a duplicate EGFR blot in Figure 5B of [13].
- Figure 8B and 8E: The western blots were reused inFigure 8D of [7] and 10A of [14] in the 2016 articles by the same authors [7].
- Supplementary Figures 1A and 4C: The western blots for JARID1B, H3K4me1 and H3 are the duplicates of p-Akt blot in Figure 6B of [2], EGFR in Figure 5B of [13] and Figure 1C b-actin of [10].

The authors did not respond to requests to comment on the concerns and provide the raw data. Due to the substantial number of duplications and overlaps in the manuscript, and the absence of original supporting data, the Editorial decision has been made to retract this paper. Oncotarget has reached out to all authors to confirm this retraction, but received no response.

Original article: Oncotarget. 2015; 6:12723–12739. 12723-12739. https://doi.org/10.18632/oncotarget.3713
